# A Comparative View on Molecular Alterations and Potential Therapeutic Strategies for Canine Oral Melanoma

**DOI:** 10.3390/vetsci8110286

**Published:** 2021-11-22

**Authors:** Laura Hardwick

**Affiliations:** Wellcome Sanger Institute, Wellcome Genome Campus, Hinxton, Cambridge CB10 1SA, UK; lh28@sanger.ac.uk

**Keywords:** canine, genetics, melanoma, malignant, oral, pathogenesis

## Abstract

Canine oral melanoma (COM) is a highly aggressive tumour associated with poor prognosis due to metastasis and resistance to conventional anti-cancer therapies. As with human mucosal melanoma, the mutational landscape is predominated by copy number aberrations and chromosomal structural variants, but differences in study cohorts and/or tumour heterogeneity can lead to discordant results regarding the nature of specific genes affected. This review discusses somatic molecular alterations in COM that result from single nucleotide variations, copy number changes, chromosomal rearrangements, and/or dysregulation of small non-coding RNAs. A cross-species comparison highlights notable recurrent aberrations, and functionally grouping dysregulated proteins reveals unifying biological pathways that may be critical for oncogenesis and metastasis. Finally, potential therapeutic strategies are considered to target these pathways in canine patients, and the benefits of collaboration between science, medical, and veterinary communities are emphasised.

## 1. Introduction

Canine melanocytic neoplasms most frequently arise in the oral cavity and mucus membranes of the lips (79%), with cutaneous tumours (11%), digital or subungual (8%), and other sites (2%) being less common. Together, they account for around 7% of all canine malignant neoplasms, forming the leading malignancy (35%) of the oral cavity [1]. Anatomical location influences tumour behaviour, with metastasis in up to 97% of oral melanomas, 100% of subungual, and 84% of digital tumours [2], whereas cutaneous and ocular melanomas are predominantly benign [1].

This review focuses on canine oral melanoma (COM), a common diagnosis, particularly in older dogs, and carrying a grave prognosis. There is no clear consensus regarding canine breeds predisposed to oral melanoma, with some studies highlighting breeds such as Poodles [2], Cocker Spaniels, and Golden Retrievers [3], while others find no overall breed predilection [4]. In general, affected dogs have more heavily pigmented skin and oral mucosa, but no predisposing germline alleles have been identified [5,6], and it is not clear whether over-representation of breeds simply reflects their prevalence in the population studied [7]. COMs are generally characterised by local invasion, rapid progression, high metastatic propensity, and frequent recurrence [2,8]. While there are a subset of histologically well-differentiated oral melanocytic tumours [9], over 90% are diagnosed as malignant on biopsy [8,10]. Current treatment modalities include radical surgical excision, radiotherapy, and/or systemic chemotherapy, but frequently with disappointing results [8,11]. Median survival times for World Health Organisation (WHO) stage II and III disease are often around 200–250 days [11,12,13,14], and survival of dogs with pulmonary metastasis at the time of diagnosis is less than two months [15]. Immunotherapy is a new and developing modality [16], but clearly, there is an urgent need for novel therapeutic strategies.

Cancer is a leading cause of death in dogs [17], so could we benefit from joining forces with our human medic counterparts in a “One Health” approach? Perhaps reflecting common physiology, lifestyle, and environmental exposure to carcinogens, the range of spontaneously occurring tumours in dogs and humans display striking similarities, and these tumours arise and evolve in the presence of an intact immune system [18,19]. Much more is known about the molecular pathogenesis of human cancers, and this provides veterinary medicine with candidate factors for targeted investigation. Furthermore, veterinary medicine is now entering a new era of genomic and transcriptomic research, facilitating direct collaboration to develop innovative cancer therapies [20]. Owners may access state-of-the-art medicine for their beloved pets, and in return, canine patients present an unrivalled natural disease model to expedite the translation of new drugs to human patients [11].

The aim of this review is to present our current understanding of the molecular alterations that underlie COM. This is a highly heterogenous disease, thus two patients may have the same histological diagnosis while having different underlying molecular changes [21]. As such, it is useful to take a broader view to determine the overarching cellular pathways and functions that are critically deregulated during oncogenesis. Comparison with the molecular pathogenesis in human mucosal and canine cutaneous melanoma can give further insight into fundamental aspects of melanoma biology, providing the rationale for future targeted therapeutics.

## 2. Methods Available to Study Molecular Alterations in COM

It is firstly useful to consider different methods and technologies used in canine melanoma research, particularly with respect to the strengths and limitations of various approaches. When faced with inconsistencies between studies, it is important to consider that these may be due to differences between patient cohorts, tumour location/stages, sample processing, and sequencing platforms, in addition to factors such as intrinsic tumour heterogeneity and passenger mutations arising secondary to genetic instability.

### 2.1. DNA Analysis

For veterinary genomics, comparative genomic hybridisation (CGH) and array-based CGH are the most commonly used technologies at present, along with fluorescence in situ hybridisation (FISH), quantitative PCR, and DNA microarrays, as well as targeted, whole exome, or whole genome sequencing [20]. FISH utilises fluorescently labelled probes that are hybridised to specific regions of the test genome in order to visualise chromosomal numbers. This can be performed where there is limited sample material available, but only specific genomic loci are evaluated depending on the probes used. By comparison, CGH scans the entire genome for copy number aberrations (CNAs) by comparing the intensity of fluorescence from differentially labelled test and control genomes hybridised onto normal metaphase chromosomes. In modern array CGH (aCGH), enhanced specificity is achieved by hybridising the test and reference DNAs with a spotted array of short oligonucleotide probes that together span the entire genome. This has an added advantage of being compatible with DNA extracted from formalin-fixed paraffin embedded tissues [22]. A similar principle of hybridisation is used for sequence detection with massively parallel arrays of known oligonucleotide probes that are immobilised on a chemical matrix. High automation and reproducible results are achieved with microarrays, but sequence detection is restricted by the characteristics of probes used. In the new era of high-throughput next generation or deep sequencing, whole genomes or exomes can be explored in an unbiased way for the detection of novel sequence variants [23].

### 2.2. RNA Analysis

A quantitative approach to determine the relative or absolute amount of messenger RNA (mRNA) encoding the protein of interest is achieved through reverse transcription polymerase chain reaction (RT-PCR). mRNA is converted into complementary DNA (cDNA), which is amplified in a PCR reaction with fluorescently labelled probes, enabling quantification of PCR amplicons (qPCR). This method can be applied at scale, from microarrays up to high-throughput automated sequencing by RNA-seq [24]. Data can, however, be limited by poor quality or degraded mRNA, for example, when using formalin-fixed paraffin embedded (FFPE) tissues [25,26].

### 2.3. Immunohistochemistry

Histological assessment remains the cornerstone of clinical diagnosis, and immunohistochemistry (IHC) can aid with both diagnosis and prognosis [27]. For example, a panel of antibodies against PNL2, Melan-A, TRP-1, and TRP-2 are sensitive and specific to determine the melanocytic origin of amelanotic tumours [28], and IHC for Ki67 as a cell proliferation marker forms a clinically useful prognostic parameter [29]. IHC has also been extensively used in research to investigate protein expression, with additional visualisation of the spatial location of the protein. The availability of validated antibodies can be restrictive and there are no universal standardised scoring methods. However, semi-quantitative schemes are often based on the number of positive cells per high-powered field, and sometimes staining intensity is also assessed [30].

### 2.4. Cell Lines

In vitro cell lines provide an invaluable tool for initial interrogation of molecular alterations, dysregulated pathways, and effects of therapeutic agents. Multiple canine melanoma cell lines have been established from primary oral and cutaneous melanomas, as well as their metastases (Table 1). These cell lines resemble the original tumours in terms of morphology, histology, karyotype, mutational landscape, and pharmacological sensitivity or resistance, illustrating the genetic and phenotypic diversity in canine melanoma [31].

A comparison of two cell lines, one from the primary tumour and the other from its metastasis, provides a unique insight into initiator events versus changes that accompany tumour progression [42]. Furthermore, cell lines from malignant oral tumours have recently been used to demonstrate the presence of cancer stem cells. These quiescent cells are enriched with drug efflux pumps that confer therapeutic resistance, and phenotype switching to a proliferative state may underlie clinical tumour recurrence [35].

However, despite the preclinical utility of in vitro cell cultures, they do not recapitulate the complex network of tumour-associated cells and secreted molecules that constitute the tumour microenvironment in vivo. In this way, tumour cells are not subject to the bidirectional communication with stromal and immune cells [43], and cells are cultured in normoxyic incubators that do not reflect hypoxic selection pressure within solid tumours [21]. Similarly, xenograft models with subcutaneous transplantation of tumours into rodent models enable preclinical study of drug toxicity, but recipients are necessarily immune deficient, and thus tumour growth is not in the context of the immune microenvironment. As such, drugs may perform well in preclinical studies, but lack efficacy in subsequent clinical trials [39,44].

## 3. Mutational Landscape in Canine Oral Melanoma

Multiple parallels exist between the mutational landscape in canine oral melanomas and human mucosal and acral subtypes. These tumours are characterised by dramatic chromosomal rearrangements and fewer single nucleotide variations (SNVs), while lacking the classic UV-induced signature seen in human cutaneous tumours [3]. Interestingly, canine cutaneous melanocytic tumours share recurrent aberrations that are distinct from melanomas arising in the oral cavity [45], and the frequency of specific point mutations varies between oral and acral locations [3]. This is not only important to explore molecular mechanisms in tumour subtypes with different biologic behaviours, but also highlights the need to specify tumour location when reporting sequencing data.

### 3.1. Chromosomal Rearrangements

Early cytogenetic analysis revealed the substantial karyotype instability in COM [46], and this can lead to complex genome-wide profiles by array CGH [45]. Determining the critical driver alterations is thus complicated by passenger events, but genetic alterations that show recurrent and unidirectional changes are more likely to contribute to functional pathogenesis, particularly when conserved in tumours from different species [5].

At a chromosome level, COMs are associated with whole gains affecting Canis familiaris (CFA) chromosomes 13, 17, 20, 29, and 36, with whole losses affecting CFA2, 22, and 27, together with focal losses and gains on CFA10 and 30 [3,5,45,47,48]. These latter sigmoidal aberrations are suggestive of major structural changes that may occur through chromothripsis, with chromosome shattering and re-fusion, resulting in a region of duplication followed immediately by a region of loss [3,45]. In a qPCR-based study of 73 COMs, focal amplifications were detected on CFA10 and 30 in 49.3% and 50.7% of cases, respectively, with 72% of dogs having at least one of these aberrations [49]. The sigmoidal profile of CFA30 is one of the most recurrent features in COM, present in up to 60% of tumours and detected in COM cell lines [3,5,31,45]. Focal CFA30 amplification is significantly associated with a subset of tumours with high mitotic index, amelanocytic phenotype, and reduced overall survival time [49]. Given the noticeable absence of this signature in cutaneous tumours, the complex CFA30 profile may directly contribute to aggressive tumour behaviour [45], particularly as a similar profile is found on the orthologous human chromosome HSA15 in human acral melanomas [5,45].

### 3.2. Copy Number Aberrations

Mechanistically, these chromosomal changes can result in copy number aberrations (CNA) of genes within the region affected, and deregulation of some of these genes can be critical to the molecular pathogenesis of COM. For example, within the affected region on CFA30, the mitogen activated protein kinase (MAPK) signalling pathway can be enhanced through the loss of *Spread1* and amplification of *Trmp7*, which results in 20-fold higher transcript abundance [5,48]. This *Trmp7* CNA is reported in 8/10 COM tumours in one study, with one dog also having the amplification present in circulating tumour DNA [50]. Other genes that are lost on CFA30 include two mitotic regulators involved in chromosome segregation, namely *Knstrn* and *Bub1b* [5,47,48], with significantly lower *Bub1b* transcripts detected in melanoma tumours compared with matched normal tissue [51].

With respect to CFA10, *Mdm2* is recurrently amplified, with a fourfold enhancement of transcripts across a cohort of 39 oral melanoma tumours compared with adjacent normal tissue [51], and up to 19-fold enhancement for individual tumours harbouring the CNA [48]. Focal amplifications of *Mdm2* are reported in 30–50% of COMs, statistically associated with *Cdk4* copy number gains, and being mutually exclusive with inactivating mutations in *Tp53* [3,47,48,49,50]. Interestingly, in the absence of *Mdm2* amplification, alternative copy number increases are detected in *Mdm2 binding protein* and *Tp53 binding protein* [47]. As MDM2 protein is a negative regulator of TP53 protein, each of these aberrations would contribute to perturbation of the p53 pathway and release of cell cycle inhibition.

Another commonly deleted gene is that of the cyclin-dependent kinase inhibitor 2A (*Cdkn2a*) gene on CFA11, which includes the *Ink4* locus encoding tumour suppressor protein and cell cycle inhibitor *p16*. Homozygous loss of *Cdkn2a* has been found in 3–68% of COMs, including both treatment naïve tumours, and as an additional mutation at the time of disease progression [3,45,48]. Similarly, Retinoblastoma susceptibility (RB1) protein is a critical tumour suppressor that regulates the G1-S phase transition in the cell cycle. Unidirectional loss of the *Rb1* gene on CFA22 is detected in up to 35% of COMs, including two primary tumours used to derive cell lines Ocr_OCMM1X and Ocr_OCMM2X [31,45,47].

### 3.3. Recurrent Single Nucleotide Variants

Canine sequencing studies range from the investigation of targeted exons to genome-wide approaches, and while the mutational burden in COM is low, ranging from 1.8 mutations per megabase to 4.9 on disease progression [48], there are notably recurrent genes affected in hotspots conserved across species [5]. For example, gain of function mutations of NRAS family members contribute to constitutive activity in the MAPK signalling pathway, with codon 61 mutation occurring in 13–30% of human melanomas [52]. Corresponding canine NRAS Q61 mutations are reported in 2–7% of COMs [2,39], with other recurrent mutations at NRAS G12 [5]. Similarly, a conserved mutation hotspot occurs in the canine and human *Pten* gene [2], and a significant reduction in PTEN protein expression is seen in melanomas from different anatomical locations [53].

A range of truncating and missense mutations have been detected in *Tp53* in 8–19% of canine melanomas and, as mentioned above, these are mutually exclusive with *Mdm2* amplification, and frequently occur in the absence of *Ras* mutations [3,5]. Further whole genome/exome studies are likely to reveal additional mutations and their functional redundancy to dysregulate critical cellular processes. This is also likely to clarify the role of genes such as putative tumour suppressor gene (*Ptprj*), which is the most commonly mutated gene in 7/31 mucosal melanomas in one study [3], but mutated in only 2/65 in a subsequent study [5].

### 3.4. Notable Genes Lacking Recurrent Aberrations in COM

Taking a candidate gene approach by extrapolation from human studies, there are genes that are frequently mutated in human cutaneous melanomas that notably lack alteration in COM and human mucosal subtypes. For example, activating mutations at BRAF codon 600 occur in up to 60% of UV-induced human melanomas, but COMs and derived cell lines are consistently BRAF wild-type [2,3,39,48,54,55]. Similarly, *Ccnd1* encodes for cell cycle protein CYCLIN D1, but only single nucleotide polymorphisms with no functional effect have been detected in COM [56].

The proto-oncogene *Kit* encodes a tyrosine kinase receptor for stem cell factor. Activating mutations in KIT protein have been detected in human melanoma and up to 30% of canine mast cell tumours, resulting in constitutive ligand-independent signalling activity, and providing a rationale for therapeutic use of tyrosine kinase inhibitors (TKIs) [57]. However, the role of KIT in COM is still unclear. KIT is expressed during normal melanocyte development, and variable degrees of immunostaining are reported in 50–85% of COMs [58,59,60]. Copy number increases in *Kit* have been reported in 26–65% of COMs, either as focal amplifications or part of large chromosomal rearrangements [3,45,60], but these do not necessarily result in increased expression at a protein level [60], and there is no significant correlation between KIT expression and any histopathologic feature, WHO stage, or overall patient survival times [61,62]. A recent clinical trial of TKI masitinib mesylate in 17 patients with advanced COM yielded disappointing results [63], and further trials are required to assess TKI use as adjunctive treatments, particularly for the individual cases where activating mutations are present [3,59,64].

## 4. Epigenetic Alterations in Canine Oral Melanoma

An addition level of complexity is added through the dynamics of the epigenome, with gene expression regulated by a variety of reversible modifications of DNA or histones, and translation of mRNA influenced by a diverse array of non-coding RNAs [65]. One such epigenetic mark is DNA methylation of cytosine residues in CpG dinucleotides. These sites are found in repetitive elements throughout the genome, and clustered in so-called CpG islands (CGI), which are present in the promotor regions of around half of all mammalian genes [66]. While the former are usually methylated, CGIs are frequently unmethylated, and within promoters, methylation can lead to gene silencing, either through a steric interference with transcription machinery, or by recruiting chromatin remodelling factors [65]. A recent canine study has taken an IHC approach with a monoclonal antibody to 5-methyl-cytosine, revealing predominantly weak staining or negative nuclei in COMs, but staining intensity in normal melanocytes was not determined, and gene-specific methylation was not determined [67]. Alternative next generation sequencing methods illustrate that, as with various human cancers, the genome-wide methylation signature in COM shows a reversal of the normal pattern, with tumours showing widespread hyper-methylation in CGIs and relative hypo-methylation at non-CGI sites [68]. Thus, promoter methylation in tumours can result in silencing of critical tumour suppressor genes, and this has been demonstrated with methylation of both DNA and histones in the promotor region of the gene encoding cytokine TNF-α in melanoma cell lines [69]. In contrast, the repetitive non-CGI element known as long interspersed nucleotide element1 (*Line1*) is 74–76% methylated in normal canine mucosa, but this is reduced to 58–64% in canine melanoma cell lines and COM tumours [70].

In terms of histone modifications, epigenetic marks such as H3K4 are associated with gene activation, while H3K9 or H3K27 correlate with transcriptional repression. The JARID1/KDM5 family of histone demethylase enzymes selectively targets and erases the activating epigenetic marks of di and tri-methylated H3K4. Over-expression of JARID1B has been reported in COM tissues, and in vitro use of histone demethylase inhibitors produces growth inhibitory effects when used alone, even in cisplastin-resistant cell lines [71]. Taken together, epigenetic dysregulation may contribute to the molecular pathogenesis of COM, opening a therapeutic opportunity if selected epigenetic marks could be reversed.

## 5. MicroRNAs

Non-coding RNAs (ncRNAs) are a highly diverse collection of RNAs that function in almost all aspects of cell biology, with dysregulation of ncRNAs providing another potential oncogenic alteration [72]. Transcriptome analysis of total RNA indicates that ncRNAs are twice as abundant in COM tissue compared with matched controls [73], with differentially expressed ncRNAs having a higher tissue specificity for diagnostic purposes than changes in mRNA profiles [51].

The best characterised ncRNAs are the small 19–25 nucleotide microRNAs (miRNAs), which are promising candidates for diagnosis, prognosis, and novel therapies in COM [74,75,76,77]. Animal miRNAs typically have only partial complementarity to the 3′ untranslated region of their target mRNAs, enabling each miRNA to have a broad range of potential targets for translational repression [78]. This may result in oncogenic effects with negative regulation of critical tumour suppressor genes, or conversely, anti-cancer effects from negative regulation of cellular oncogenes. Various studies have identified an overlap in the miRNomes between human and canine melanomas, identifying dysregulated miRNAs in canine oral melanoma [73,74,77,79,80], canine cutaneous melanoma [43,80], and canine uveal melanoma [26]. The function of differentially expressed miRNAs is not always known, but selected examples are presented below.

### 5.1. MicroRNA-203

MiRNA-203 is a tumour suppressor transcript that is significantly reduced in COM relative to normal tissue, forming a potential prognostic factor with the miRNA-203 level inversely associated with tumour stage and overall survival time [73,77,81]. Mechanistically, downregulation results from methylation of CpG islands in the DNA upstream of the miRNA-203 gene [82], and loss of miRNA-203 function in turn leads to cell proliferation via an increase in target transcripts including cell cycle component E2F3 [83] and transcription factor CREB1 [84].

### 5.2. MicroRNA-205

Another tumour suppressor transcript that is downregulated in COM is miRNA-205 [73,74], which exerts growth inhibitory and anti-migration effects by repressing EGFR superfamily member ERBB3 [77] and zinc-finger E-box binding homeobox 2 (ZEB2) [85], respectively. As a step towards utilising miRNA-205 for clinical application, a chemically modified synthetic transcript has been designed and optimised, with an aromatic benzene-pyridine analogue to provide resistance against RNA nucleases, and altered passenger sequence to enhance tumour suppressor activity [86]. Following promising preclinical results, 10 dogs received intratumoural injection of synthetic miRNA-205 after surgery or radiotherapy. These included two dogs with stage I, four dogs with stage II, three dogs with stage III, and one dog with stage IV disease. No adverse effects were reported with complete response achieved in 50% and static disease in 30%; two dogs with progressive disease started with stage II and IV COM. Overall, these results warrant further development of miRNA-based therapy [76].

### 5.3. MicroRNA-145

In canine melanomas arising from both oral and cutaneous locations, a conserved oncosuppressor role has been ascribed to miRNA-145, potentially mediated through translational repression of oncogenes such as c-MYC [79,80]. Anti-tumour effects of miRNA-145-5p in human melanoma cell lines and xenograft tumours involve targeting NRAS transcripts to suppress cell proliferation mediated by the MAPK pathway. As these effects have only been observed in cells with wild-type BRAF [75], and as the majority of COMs have no BRAF mutations [54], miRNA-145 may be a promising target in COM to counteract hyperactivity in the MAPK signalling path.

### 5.4. Circulating and Exosomal MicroRNAs as Biomarkers in Plasma

Tumour cells can also release miRNAs in extracellular vesicles, with circulating tumour miRNAs in the blood providing potential biomarkers that could be assayed by relatively non-invasive methods for diagnosis, prognosis, and monitoring response to treatment. In this respect, canine miRNA-126 has been shown to be prognostic in melanoma, adenocarcinoma, and other epithelial tumours [87], with miRNA-143 also increased in COM patient samples compared with controls, and miRNA-221 showing a gradual increase with tumour progression [88].

### 5.5. Hypoxia-Induced MicroRNAs

An essential element to consider in the tumour microenvironment is that of tumour hypoxia, with varying oxygen tensions present in different regions of the tumour, and creating a selection pressure for cells that can switch to a hypoxic phenotype through upregulation of hypoxia-inducible factors [21]. This also includes upregulation of a subset of hypoxia regulated miRNAs (HRMs), such as miRNA-450, miRNA-301, and miRNA-146 [89]. Based on in vitro studies with primary oral melanoma cell line KMeC and metastatic oral melanoma LMeC, miRNA-21 and miRNA-301 have been identified as key HRMs in primary and metastatic cells, respectively. Furthermore, this additional hypoxic response and the influence of tissue microenvironment may contribute to disparity in the reported differentially regulated transcripts [89].

## 6. Cellular Pathways and Processes Deregulated in Canine Oral Melanoma

In addition to identifying deregulated genes and proteins, next generation sequencing data also highlight the molecular heterogeneity in melanoma [42]. The most effective targeted therapies will be those that counteract the pathways and processes that are high-jacked by cancer cells. To this end, by considering the functional effects of each individual molecular alteration, a broader picture can be generated to identify pathways and processes with aberrant activity in COM.

### 6.1. Pro-Survival Mitogenic Pathways

Self-sufficiency of growth signals and constitutive activation of mitogenic pathways is a hallmark of cancer [90], and malignant tumours often show deregulation of tyrosine kinases that include growth factor receptors such as KIT, EGFR, PDGFR, and VEGFR [57]. Over-expression of epidermal growth factor receptors is associated with aggressive behaviour in human and canine cutaneous melanomas [91], but is not found to be amplified or prognostic in canine oral melanomas [92]. In contrast, IGF-1 [93] and PDGFR may be relevant to COM, and patients with tumours positive for both PDGFRα and PDGFRβ have significantly shorter overall survival [12].

**Figure 1:**
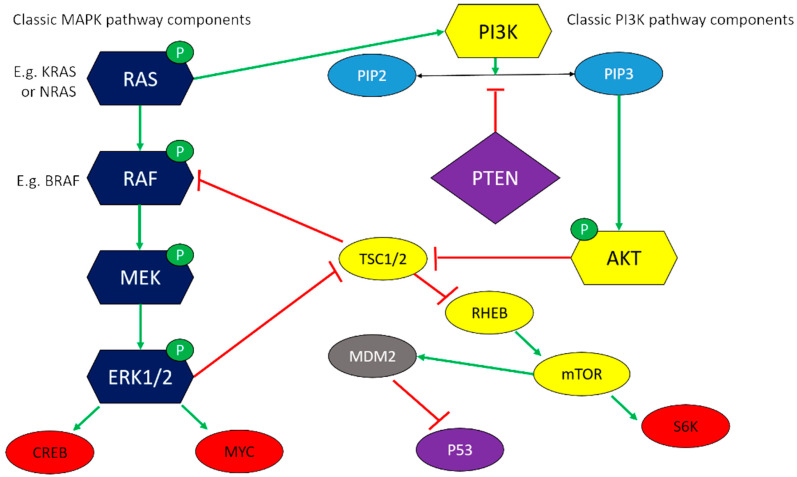
Key components of the MAPK and PI3K signalling paths featured in this review. Classical MAPK and PI3K components are shown in navy and yellow shapes, respectively. Green arrows indicate activating effects; red bars indicate inhibitory effects. Red shapes illustrate key transcription factors driving cell proliferation, growth, and survival, whereas purple shapes indicate key tumour suppressor proteins.

Two critical mitogenic cascades in melanoma are the MAPK and phosphoinositide-3-OH kinase (PI3K) signalling pathways (Figure 1). The MAPK path typically involves a cascade of phosphorylation and activation events involving RAS, RAF, MEK1/2, and ERK1/2. Nuclear translocation of phospho-ERK1/2 activates transcription factors such as MYC and CREB to upregulate genes associated with cell proliferation and survival [52]. In the PI3K path, PIP3 acts as a second messenger to activate AKT, also known as protein kinase B, with downstream targets including mTOR, a highly conserved protein that promotes protein synthesis, cell cycle progression, and angiogenesis [52]. Substantial cross-talk exists between pathways, for example, with NRAS able to activate AKT [52], but this also leads to concerns over reciprocal upregulation of one pathway upon pharmacological inhibition of the other [94].

Several pieces of evidence point to MAPK and PI3K pathways being fundamental to oral melanoma. Firstly, both canine and human tumours show immunohistochemical evidence of constitutive phospho-ERK1/2 and/or phospho-AKT in 52–95% of tumours [2,39,44,54,58,59,61,94], and this is also seen in both cell lines and xenograft models [31], with PI3K-AKT signalling influencing the phenotype of the cancer stem cell compartment [35]. Furthermore, analysis of genomic and transcriptomic data repeatedly highlights the enrichment of deregulated genes in MAPK and PI3K signalling cascades [3,43,47].

Secondly, schematic representation of these two pathways (Figure 1) also brings in familiar names from the preceding discussion on genetic and epigenetic alterations. PTEN is frequently down-regulated in canine and human melanoma [53], and a marked reduction in PTEN expression owing to negative regulation by miR-374b is also involved in acquired radioresistance of COM cell line KMeC [95]. This bifunctional phospholipid and protein phosphatase inhibits AKT activation through dephosphorylation of PIP3, and antagonises MAPK signalling through dephosphorylation of adaptor proteins in the signalling scaffold [96].

Thirdly, the functional result of aberrant activity in the MAPK and PI3K path can be achieved through redundant mechanisms. For example, BRAF is mutated in around 60% of human cutaneous melanomas, often alongside deletion of PTEN, but rarely co-existing with NRAS mutations [52], whereas in dogs, pathway activation may be achieved through loss of SPRED1 and gain of TRMP7 [45].

### 6.2. Loss of Cell Cycle Control and DNA Damage Response

The cell cycle consists of four consecutive phases (G1, S, G2, and M), with transition between phases mediated by combinations of cyclins with their respective cyclin-dependent kinases. A series of checkpoints exist, and braking mechanisms are provided by a family of cyclin-dependent kinase inhibitors (CDKIs), including p21 encoded by the *Waf-1* gene, p16 encoded by the *Ink-4a* gene, and well-known tumour suppressor p53 [52]. Cells can withdraw from the cell cycle into a state of quiescence, but in the face of cellular damage, oncogene activation, or mitogenic stress, cells can either undergo apoptosis or a stable form of cell cycle arrest called cellular senescence [52]. Thus, in addition to self-sufficiency of growth signals provided by constitutive activation of mitogenic pathways, cancer cells also need resistance to anti-growth signals [90], and this can be achieved through loss of function in cell cycle inhibitors.

The canine p53 family of proteins is comparable to humans, with p53 having pivotal roles to maintain DNA integrity, initiate DNA repair, and influence cell fate in apoptosis and senescence pathways [53,97]. Aberrations of the p53 pathway occur in cutaneous and oral melanomas in both dogs and humans, for example, by focal amplification of p53 inhibitor MDM2, increases in p53 binding proteins, truncating mutations in the p53 protein, or mislocalisation of wild-type p53 protein with nuclear exclusion [3,47,48,53].

Canine p21 is expressed in two isoforms [97], and no mutations have been reported in the *Waf-1* gene in COM, but nuclear exclusion and/or functional loss may be relevant in a subset of cutaneous melanomas [38,53]. In contrast, inactivation of p16/Rb function is a critical event in both oral and cutaneous melanoma tumours [53]. Loss of the *Rb1* gene on CFA22 occurs in up to 35% of COMs [45,47], and transcripts from the *Ink4a* gene including p16 are frequently missing or harbour in-frame deletions in canine melanoma cell lines. Taken together, these defects are likely to disable the G1 checkpoint to allow proliferation of cells even in the presence of anti-growth signals and, combined with the loss of mitosis-related proteins such as KNSTRN, BUB1, and TACC3, this may drive further large-scale chromosomal abnormalities [5,45,47].

### 6.3. Molecular Deregulation Promoting Metastasis

A critical step for tumour invasion is epithelial to mesenchymal transition (EMT), whereby epithelial cells undergo a phenotypic change, losing cell cohesion and expressing new mesenchymal proteins that can degrade extracellular matrix and promote cell motility [98]. Melanocytes are derived from neural crest progenitor cells and, during embryogenesis, physiological EMT enables these progenitor cells to delaminate from the neural tube and migrate through the mesenchyme to the basal layer of epithelia [1]. Melanocytic malignancies recapitulate this invasive phenotype as part of the multistep process to tumour metastasis, and defining the underlying molecular changes can thus open opportunities for therapeutic intervention.

#### 6.3.1. Cadherin Switching

Normal melanocytes form adherens junctions through E-CADHERIN molecules, and destruction of cellular cohesion in EMT is seen in malignant oral tumours with downregulation of E-CADHERIN and SYNDECAN1 at the transcript and protein levels [99,100]. In addition to effects on tissue architecture via loss of adherens junctions, reduced expression of E-CADHERIN leads to the disruption of normal tethering of β-CATENIN at the cell membrane. Cytoplasmic levels of free β-CATENIN are usually kept low by phosphorylation, ubiquitination, and degradation, but 87% of COMs in one study had cytoplasmic rather than membranous immunostaining [101]. Free β-CATENIN may translocate to the nucleus to activate transcription of WNT pathway genes, but the concurrent lack of nuclear staining indicates that canonical WNT/β-CATENIN signalling is not active in COM [102], so the biological effect of this cytoplasmic accumulation of β-CATENIN is still to be determined.

#### 6.3.2. Changes in Cell Motility

Migration of neoplastic cells requires breakdown of the extracellular matrix (ECM), intrinsic cell motility, and interaction with ECM proteins. As such, changes in expression of proteases and cell surface markers are potential prognostic factors to indicate invasive behaviour and metastatic potential. For example, matrix metalloproteases (MMPs) contribute to remodelling of ECM, and MMP2 is expressed at high levels in late-stage COMs and primary oral melanoma cell lines [98,100]. Similarly, FASCIN-1 is an actin-bundling protein associated with actin structures in highly motile cells. FASCIN-1 is expressed in 98% of COMs across stages I–IV, with a significant increase in FASCIN-1 IHC score in stage IV relative to stage II tumours. Localisation of FASCIN-1 particularly at the tumour periphery is supportive of a role in invasion, and as median survival time for patients with strongly immunopositive tumours is significantly shorter than those with weak or moderate staining intensity, the use of FASCIN-1 inhibitors may have clinical utility [103].

Another protein involved in embryonic EMT is Podoplanin (PDPN), a transmembrane sialoglycoprotein expressed by normal cells such as lymphatic endothelia and renal podocytes. In 80–90% of COMs, the majority of neoplastic cells display membranous expression of PDPN, and this is significantly associated with proliferative marker and negative prognostic factor Ki-67 [104]. It is not yet clear how PDPN contributes to an aggressive tumour phenotype, but knockdown of PDPN in oral melanoma cell lines inhibits cell migration and proliferation, while promoting tumour cell apoptosis [104]. Potential targeting of PDPN for therapeutic inhibition is facilitated by its location at the cell membrane, similar to glycoprotein CD44, the main cell surface receptor for ECM component hyaluronan (HA). Reminiscent to FASCIN-1, CD44 expression is significantly higher, and localised to the tumour margins in malignant oral melanomas compared with benign cutaneous tumours. Based on in vitro studies, oral melanoma cells expressing high levels of CD44 also produce substantial amounts of Hyaluronan and Versican, forming a pericellular matrix that may assist in cell migration [105].

#### 6.3.3. Angiogenesis

Angiogenesis is a further hallmark of malignancy [90], and pathway analysis identifies angiogenesis-related genes to be deregulated in COM [47]. A well-known driver of neovascularisation is vascular endothelial growth factor (VEGF), a heparin glycoprotein that binds to receptors VEGFR1 and VEGFR2 on endothelial cells. Up to 95% of COMs express VEGF [13], with VEGF secretion recorded from a canine oral melanoma cell line [106], and genetic copy number imbalances in *Vegfr2* also documented [47]. Plasma levels of angiogenic factors such as VEGF are a negative prognostic factor in human cutaneous melanoma patients, and both serum and plasma levels of VEGF are significantly elevated in COM patients relative to geriatric control dogs. VEGF levels rise with disease progression from stage I to stage IV and correlate with reduced survival time [13]. Thus, serum or plasma levels of VEGF may provide a relatively non-invasive prognostic factor for monitoring disease progression.

### 6.4. Deregulation of the Immune Microenvironment

Dynamic interplay with the host immune response shapes the growth and evolution of a tumour, described by the three phases of “immunoediting”: initial elimination of tumour cells by immune components such as cytotoxic CD8+ T cells, followed by an equilibrium stage when tumour growth is held static, and finally immune escape when the host immune response is supressed or becomes tolerant to tumour antigens [107]. It is now recognised in COM and other neoplasms that the immune profile of a tumour can provide additional prognostic information [108], and complex deregulation of immune system pathways contributes to molecular pathogenesis [48].

#### 6.4.1. Cyclooxygenase-2

Cyclooxygenase (COX) enzymes catalyse the rate-limiting step in the generation of prostanoids from arachidonic acid. COX1 is constitutively expressed in many tissues including normal skin and mucosa, whereas the COX2 isoform is absent from these normal tissues and is instead induced by pro-inflammatory and tumour promoting stimuli. Up to 100% of oral melanomas express COX2, with the highest immunolabelling at the infiltrating border of the tumour in contact with COX2 positive inflammatory cells [109]. Furthermore, the mean concentration of COX2 product prostaglandin E2 (PGE2) is elevated approximately threefold in COM compared with normal mucosa [110].

In human cutaneous melanomas, COX2 has been proposed as a diagnostic IHC marker to differentiate early lesions from benign melanocytomas [111], and in canine cutaneous and oral melanomas, COX2 may be an independent predictive marker for overall survival [112]. COX2 expression is higher in biologically aggressive canine melanomas from the digit and oral cavity compared with more benign cutaneous tumours [109,112], and high tumour cell expression of COX2 correlates with tumour recurrence and significantly shorter survival times [112].

In vitro studies indicate that COX2 induction may be stimulated by tumour necrosis factor-alpha stimulated protein 6 (TSG-6) released from adipose tissue derived mesenchymal stem/stromal cells (ADSCs) [113], or by cytokine interleukin-1 (IL-1) in the tumour microenvironment acting as a paracrine or autocrine factor to upregulate COX expression via canonical NF-κB signalling [114]. In this respect, it is interesting to note that, among the differentially expressed genes (DEGs) in transcriptome analysis of canine oral melanomas compared with normal mucosa, NF-κB transcription site binding motifs are most frequently seen among upregulated DEGs [42].

The mechanisms by which COX2 contributes to biologic behaviour of a range of tumours are not fully understood, but suggestions include a role in angiogenesis [115], upregulation of drug efflux pumps to establish multi-drug resistance [113], and a possible influence on macrophage phenotype with more pro-tumoural M2 macrophages in the presence of high COX2 [116]. However, expression of COX2 and PGE2 across different tumour cell lines does not correlate with their relative sensitivity to non-steroidal anti-inflammatory drugs (NSAIDs), and in vitro growth inhibitory effects of COX-2 selective NSAIDs generally require high concentrations and may be COX-independent [117,118]. Taken together, a greater understanding of the role of COX2 in melanoma pathogenesis is required before this can be translated into a therapeutic target, but COX2 expression by tumour cells can nevertheless provide valuable diagnostic and prognostic information.

#### 6.4.2. Tumour Infiltrating Lymphocytes and Mechanisms of Immune Tolerance

**Figure 2:**
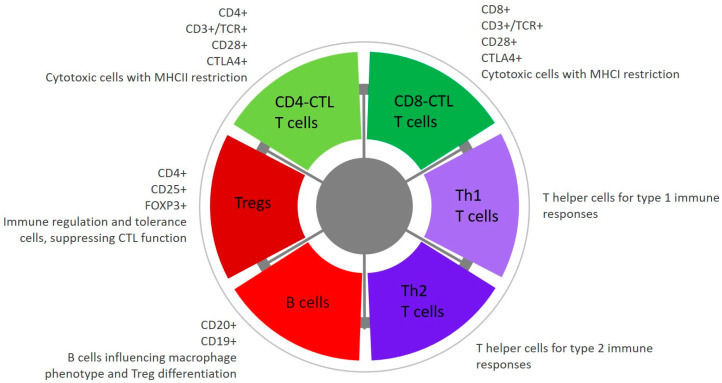
Subtypes of lymphocytes involved in immune responses to melanoma cells. Neoplastic cells are killed by cytotoxic T lymphocytes (CTLs), with anti-cancer responses supported by T helper (Th) cells. Immunosupressive and pro-tumour roles are ascribed to T regulatory cells (Tregs) and a subtype of B lymphocytes.

Tumour infiltrating lymphocytes (TILs) are a highly heterogenous collection of different subtypes of lymphocytes that infiltrate in response to tumour cells, and the balance between these different subtypes is critical to the effectiveness of the anti-tumour immune response (Figure 2). On one hand, CD4+ T helper cells can enhance the function of antigen presenting cells (APCs) and stimulate recruitment of cytotoxic CD8+ lymphocytes (CTLs) to eliminate tumour cells. Yet, on the other hand, regulatory CD4+/CD25+/FOXP3+ Treg cells promote immune tolerance by suppressing the function and cytokine release from other lymphocytes, and CD20+ B cells may have detrimental effects by influencing an M2 macrophage phenotype and promoting differentiation of Tregs [108,119,120,121]. Thus, while human and canine melanomas are considered to be highly immunogenic tumours, there is a balancing act between the different subtypes of TILs and various mechanisms by which the tumour microenvironment becomes immunosuppressive.

Firstly, tumour cells can produce inhibitory cytokines such as transforming growth factor beta 1 (TGF-β1) in the tumour microenvironment, and TGF-β1 protein is found to be elevated in the serum of dogs with metastatic oral melanoma relative to healthy control dogs [122]. TGF-β1 results in an increase in the percentage of immune suppressive FOXP3+ Tregs, and direct inhibition of CTLs, evidenced by reduced release of IL-2, IFN-γ, and TNF-α from healthy peripheral blood mononuclear cells cultured in the presence of TGF-β1. Targeted inhibition of TGF-β1 could, therefore, help to push the balance in favour of anti-cancer CTL activity, with in vitro proof of principle achieved using an immunoglobulin decoy receptor TGF-βRII [122].

This leads us to a second fundamental mechanism of immune suppression, with recruitment or selective expansion of the inhibitory Treg population. Flow immunophenotyping of TILs from treatment naïve COMs demonstrates that a high number of TILs with a low proportion of Tregs is associated with less aggressive tumour behaviour and longer patient survival [119], with similar results reported for the lymphocyte profile in peripheral blood [121]. Furthermore, when evaluating TILs microscopically, the number of FOXP3+ lymphocytes per high power field is an independent prognostic factor, with the percentage of FOXP3+ lymphocytes correlating with the mitotic count and presence of metastasis [4]. Additionally, neoplastic cells express FOXP3 in 61% of COMs and may functionally mimic Tregs [4].

The next question to address is what drives the expansion of the Treg population within COMs: one possibility is that Tregs may migrate from the circulation [123], and/or the tumour microenvironment may provide a niche for local proliferation of Tregs, for example, through the high local concentration of factors such as TGF-β1 [122]. Another interesting avenue to explore is that of the kynurenine pathway and cytosolic enzyme Indoleamine 2, 3-dioxygenase (IDO) involved in tryptophan metabolism. Depletion of tryptophan and build-up of kynurenine pathway metabolites promotes differentiation of FOXP3+ Tregs, and the mean number of IDO+ inflammatory cells in COMs is another independent factor positively associated with risk of death [4,25]. An associated tryptophan metabolism enzyme kynurenine 3-monoxygenase (KMO) is elevated in 65% of COMs and correlates with increased levels of activated transcription factor pSTAT3, which may drive expression of IDO [124]. Prognostic threshold values for IDO+, FOXP3+, and KMO+ cells have been determined with varying sensitivity and specificity [4,25,124], and further investigation may reveal the mechanisms linking tryptophan metabolism to Treg differentiation with a view to manipulating the TIL subtype for clinical benefit.

A third mechanism of immune escape is through high-jacking of co-inhibitory immune checkpoint receptors. For example, cytotoxic T lymphocyte antigen (CTLA4) is expressed on both activated lymphocytes and Tregs, binding B7 ligands on APCs. This checkpoint becomes active in the face of persistent immune stimulation, resulting in inhibition of T cell proliferation and preventing engagement of the T cell-stimulating receptor CD28 to B7 ligands [125]. Another checkpoint inhibitor that is gaining significant attention is programmed death 1 (PD-1), expressed by activated lymphocytes to suppress T cell cytokine production when stimulated by binding to programmed death ligand 1 (PD-L1). This is a physiological mechanism of peripheral tolerance, and a regulatory feedback loop following T cell activation [16]. Ninety to 100% of COMs express PD-L1 [126,127,128], possibly in response to IFNγ [126] and modulated by interaction with CD3+ T cells [120], with membrane expression maintained by CMTM6 (CKLF-like MARVEL transmembrane domain protein 6) [129]. Tumour cell expression of PD-L1 is a negative prognostic indicator in human oncology, with similar findings established for PD-L1 expression in canine malignant mammary gland tumours [130]. Critically, PD-1 mediated immune suppression is reversible, with promising clinical application of monoclonal antibodies to block the interaction between PD-1 and PD-L1 [128].

## 7. Potential Therapeutic Targets and Future Directions

COM remains a challenging disease in veterinary medicine, complicated by both interpatient and intratumour heterogeneity, giving rise to varied responses to conventional therapy and rapid selection of resistant tumour clones [5]. Precision medicine is now at the forefront of human oncology, whereby treatment is selected to achieve maximum benefit for the patient, while minimising the personal and financial costs associated with ineffective treatments [21]. This concept is equally desirable for our veterinary patients, aiming for targeted therapies to disrupt dysregulated pathways that are unique to cancer cell biology, thereby reducing systemic side effects [36]. This necessitates a detailed knowledge of the underlying molecular alterations in tumour biology. Some promising areas where new therapeutic options for COM may emerge are discussed below.

### 7.1. Targeting Deregulated Signalling Pathways

Hyperactivity in the MAPK and PI3K pathways is a conserved feature between canine and human melanomas, even if the underlying molecular mechanisms differ. Owing to extensive cross-talk between these two signalling cascades, inhibition of one pathway can lead to compensatory activity in the other and potential tumour resistance [39]. As in vitro proof of principle with canine melanoma cell lines of differing basal ERK and AKT activity, synergistic growth inhibitory effects are observed by combining an MEK inhibitor with a dual PI3K/mTOR inhibitor [39]. Thus, multimodal strategies may be beneficial, but so far, combination therapies in humans are limited by high toxicities, so innovative methods for specificity in targeting are required.

In this respect, an alternative strategy to disrupt oncogenic MAPK signalling in human cancers is to target scaffold protein IQGAP1, which regulates the assembly of MAPK components for pathway activation. Of particular interest is that inhibition of IQGAP1 selectively reduces aberrant rather than physiological MAPK signalling [131]. The orthologous canine IQGAP1 protein is expressed in melanoma cell lines, and shows a high degree of sequence conservation in the ERK-interacting WW domain. Furthermore, inhibition of IQGAP1 function in canine mucosal melanoma cell lines, either by CRISPR-Cas9 mediated gene knockdown or by competitive inhibition from a WW domain peptide mimetic results in growth inhibition and reduced phospho-ERK [131]. These are promising preliminary results to support this novel approach for targeting scaffold proteins in oncogenic signalling cascades.

Conversely, while targeting over-active proteins for inhibition, it can also be beneficial to increase levels of tumour suppressor proteins such as p21 to restore sensitivity to conventional chemo or radiotherapy through cellular senescence and/or apoptotic pathways. Among the different intracellular degradation pathways, p21 is targeted by the NEDD8-regulated neddylation system. Inhibition of NEDD8-activating enzyme (NAE) with micromolar concentration of Prevonedistat is able to trigger apoptosis and cellular senescence in patients with primary and metastatic COM. Theoretical concerns include the potential for cross talk and disruption of other substrates of this NEDD8 pathway, but initial phase I human clinical trial data are promising [36].

### 7.2. Reinstating Apoptosis

Evasion of apoptosis is one of the original hallmarks of cancer that additionally contributes to chemotherapeutic drug resistance [90]. Apoptosis is a means of programmed cell death, triggered by a variety of extrinsic and intrinsic stimuli that converge on members of the Bcl2 family. Pro-apoptotic members such as BH3-only proteins BIM and BAD, and multi-domain members BAX and BAK, are counteracted by anti-apoptotic multi-domain members such as BCL-2, BCL-Xl, and MCL-1. Once triggered, apoptosis is executed with permeabilisation of the mitochondrial membrane and release of cytochrome c, activation of APAF-1 (apoptotic protease activating factor 1), and a cascade of cysteine protease caspase enzymes [52,132].

In human cutaneous melanoma, evasion of apoptosis occurs through upregulation of BCL2 and/or loss of APAF-1 [52]. Enhancing pro-apoptotic pathways may sensitise tumours to systemic chemotherapeutics, and various in vitro studies have used targeted knock-down approaches with small interfering RNA (siRNA) to identify candidate proteins for which small molecule inhibitors could be developed. For example, the apoptotic rate of malignant oral melanoma cells can be enhanced by siRNA-mediated knockdown of the *Bcl2* gene [133], or through a BH3 mimetic to post-translationally inhibit BCL2 protein [134], and BCL2 inhibition synergises with carboplatin-induced cell death in vitro [134]. Clearly, these preliminary results have a long way before clinical application, but there is a rationale for adjunctive use of pro-apoptotic agents if these could be adequately targeted [134].

### 7.3. Targeting the Immune Response

Immunotherapy to harness the patient’s immune system is becoming the fourth pillar in human cancer treatment, with the main limitation being aberrant immune activation and autoimmunity. A detailed discussion is beyond the scope of this review, but suggested approaches include intratumoural injection of bacterial cell components and superantigen, or injection of cDNA encoding human recombinant granulocyte-macrophage colony stimulating factor (hr-GM-CSF) that acts as a growth factor for dendritic cells and macrophages. Similarly, support for the adaptive immune system by injection of cytokines such as human recombinant IL-2 to support expansion of effector T cells or delivery of co-stimulatory molecules such as CD40L to enhance T cell activation. Adoptive cell transfer involves infusion of tumour-specific T lymphocytes into the patient, and lymphokine-activated killer cell therapy involves non-specific in vitro stimulation of autologous peripheral blood mononuclear cells that are then returned to the patient (reviewed in [16,132,135]). At present, the relevance to canine patients is largely speculative, but two of the most promising developments are presented here; that is, targeting of immune checkpoint inhibitors and melanoma vaccination.

#### 7.3.1. Immune Checkpoint Inhibitors

Tumour cell expression of PD-L1 can supress the anti-cancer host immune response, as described above. The first monoclonal antibody to be trialled in COM patients used a chimeric rat anti-canine PD-L1 approach (c4G1) substituting in the constant regions of dog IgG to reduce immunogenicity [15]. A similar approach was taken with rat/canine chimeric antibody ch-4F12-E6, which was additionally caninised (ca-4F12-E6) with further substitution of the rat-derived resides in the complementary determining region [136]. These antibodies are generally well tolerated and show promising results to extend patient survival up to threefold longer in clinical trials with advanced stage IV COMs [128,136].

#### 7.3.2. Vaccination Strategies

Active immunotherapy aims to stimulate the host immune response to selected cancer-associated antigens by vaccination with a bacterial expression plasmid containing the gene of interest. The main challenge is to overcome self-tolerance, but this can be achieved by vaccination with an orthologous gene from another species, and cross-reactivity of the immune effector response [137]. The xenogeneic vaccine strategy with human Tyrosinase gene is employed in the licenced OnceptTM vaccine [138], with other vaccine trials including the human Glycoprotein 100 (GP 100) gene [37] and human chondroitin sulphate proteoglycan-4 (CSPG4) gene [139,140].

### 7.4. Harnessing Cancer-Associated Antigens

Selective expression of cancer-associated antigens on neoplastic cells presents a unique opportunity that can be exploited in several ways. Theoretically, neutralising monoclonal antibodies could be used to target antigens involved in cancer development, and a recent phase I/II clinical trial has taken this approach with chimeric mouse/dog anti-podoplanin antibody [141]. Alternatively, novel antibody–drug conjugates can be utilised for targeted delivery of a given chemotherapeutic agent [142]. In vivo optical imaging may also be possible using fluorescently labelled high affinity peptides that bind canine alpha-3 integrins, with potential application for the detection of early metastasis or intra-operative assessment of tumour margins [33].

While these therapeutic applications may require significant development, cancer-associated antigens could also assist in diagnostics, particularly in the case of challenging amelanotic spindle cell tumours that need to be differentiated from other spindle cell sarcomas [143]. Heterogeneity of canine melanomas is a complicating factor that will likely necessitate the use a panel of diagnostic markers, such as the immunodiagnostic cocktail of Melan-A, PNL2, TRP-1, and TRP-2 [28]. For amelanotyic subtypes, alternative markers such as CD146/MCAM and CSPG4 may also be useful [98,144,145].

## 8. Conclusions

In the last 20 years, there has been an exponential development of high throughput sequencing technologies and the resulting genome/exome/transcriptome data have given an unprecedented and unbiased insight into the molecular alterations in canine melanoma. This has facilitated the discovery of novel mutations, identified diagnostic and prognostic molecular markers, and revealed molecular pathways that are critically deregulated. The similarity between COMs and human mucosal and acral subtypes has been repeatedly demonstrated; from histopathological concordance [58], to the mutational landscape [3,5,45,47], to dysregulated signalling pathways including MAPK and PI3K [39,44,58]. This wealth of information now paves the way for the development of targeted therapies with improved clinical response and fewer side effects. Potential anti-metastasis therapeutics are already in development in human medicine [146], and canine patients may solve the problems surrounding the translational gap by providing a relevant spontaneous disease model [120]. To this end, collaborative work between the science, medical, and veterinary communities will surely strengthen our collective success.

## Figures and Tables

**Table 1:** Established canine oral melanoma cell lines.

Cell Line	Derivation	Reference
KMeC	Primary oral melanoma	[32]
LMe-C	Lymph node metastasis of oral melanoma	[32]
UCDK9M1	Skin metastasis of oral melanoma	[33]
UCDK9M2	Lymph node metastasis of oral melanoma	[33]
UCDK9M3	Primary oral melanoma	[33]
UCDK9M4	Primary oral melanoma	[33]
UCDK9M5	Lymph node metastasis of oral melanoma	[33]
CMM1	Primary oral melanoma from patient with lymph node metastasis	[34]
CMM2	Primary oral melanoma from patient with no metastasis	[34]
Ocr_OCMM1X	Xenograft derived from primary stage IV oral melanoma	[31,35]
Ocr_OCMM2X	Xenograft derived from primary stage III oral melanoma	[31,35]
CML-1	Primary oral melanoma	[36]
CML-10C2	Primary oral melanoma	[36]
17CM98	Lymph node metastasis of primary oral melanoma	[37]
TLM-1	Primary oral melanoma	[38]
JONES	Primary oral melanoma	[39]
JENNY	Primary oral melanoma	[40]
SCOOTER	Primary oral melanoma	[40]
SHADOW	Pulmonary metastasis of primary oral melanoma	[40]
CMGD-2	Primary oral melanoma	[41]
